# Dataset of total, oligomeric alpha-synuclein and hemoglobin levels in plasma in Parkinson׳s disease

**DOI:** 10.1016/j.dib.2016.11.089

**Published:** 2016-11-28

**Authors:** A. Emelyanov, P. Andoskin, S. Pchelina

**Affiliations:** a Petersburg Nuclear Physics Institute, St. Petersburg, Russia; b Pavlov First Saint Petersburg State Medical University, St. Petersburg, Russia; c St. Petersburg Academic University - Nanothecnology Research and Education Centre, RAS, St. Petersburg, Russia; d Institute of Experimental Medicine, St. Petersburg, Russia

## Abstract

This data article presents a dataset of total, oligomeric alpha-synuclein and hemoglobin levels in plasma of drug-naïve PD patients and controls. This is the first attempt to assess the effect of hemolysis rate on oligomeric alpha-synuclein levels in peripheral plasma. The data are associated with the research article “Oligomeric alpha-synuclein and glucocerebrosidase activity levels in GBA-associated Parkinson׳s disease” (Pchelina et al., 2016) [1].

**Specifications Table**

**Table:** 

Subject area	*Medicine*
More specific subject area	*Neurology, Neurodegenerative diseases*
Type of data	*Table, Graph*
How data was acquired	*Enzyme-linked immunosorbent assay (ELISA), Microplate Spectrophotometer, SPSS* 12.0
Data format	*Analysed*
Experimental factors	*Blood plasma samples were collected using centrifugation*
Experimental features	*The relationship between total, oligomeric alpha-synuclein and hemoglobin levels in plasma in Parkinson׳s disease was determined.*
Data source location	*St.Petersburg, Russian Federation*
Data accessibility	*Data is within this article*

**Value of the data**

• These data provide the correlation between oligomeric alpha-synuclein and hemoglobin plasma levels in drug-naive PD patients and controls.
• The data are of value for further experiments on the assessing of alpha-synuclein levels in peripheral blood and cerebrospinal fluid (CSF) samples.
• The data have significance due to the fact that hemolysis has effect on total but not oligomeric alpha-synuclein levels in peripheral blood plasma.

## 1 Data

**Fig. 1:**
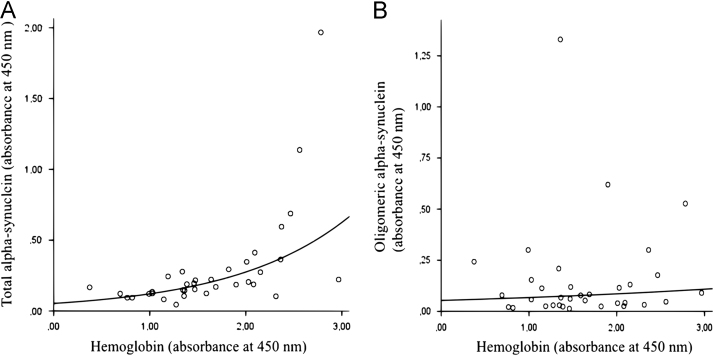
Correlation between alpha-synuclein species and hemoglobin plasma levels in combined group of PD patients and controls. (A) Positive correlation between total alpha-synuclein and hemoglobin levels (*r*=0,714; *p*<0,0001). (B) Correlation between oligomeric alpha-synuclein and hemoglobin levels (*r*=0, 198; *p*=0,254).

Significant positive correlation of total alpha-synuclein with hemoglobin levels was observed in combined group of iPD patients and controls (*r*=0,714; *p*<0,0001) and separately (for iPD patients – *r*=0,656; *p*=0,004; for controls – *r*=0,746; *p*<0,0001) (Fig. 1). However, the correlation between oligomeric alpha-synuclein and hemoglobin levels in the above mentioned groups was absent (for combined group – *r*=0,198; *p*=0,254; for iPD – *r*=0,234; *p*=0,366; for controls – *r*=0,049; *p*=0,848). We did not find any significant difference in total, oligomeric alpha-synuclein levels and oligomeric/total alpha-synuclein ratio between iPD patients and controls (Table 1) [1].

## 2 Experimental design, materials and methods

### 2.1 Sample preparation

Blood plasma samples were collected from 17 drug-naïve patients with iPD (mean age 62.44±9.67, 6 males) and age/sex-matched 18 controls (mean age 63.83±10.86, 10 males). PD patients were diagnosed with PD at Pavlov First Saint Petersburg State Medical University, Saint-Petersburg, Russia. Subjects without neurological disorders were included in the control group. The study was approved by the local ethics committee. All the participating subjects provided informed consent. Samples were collected using centrifugation at 3000 rpm at 4 °C for 20 min. All samples were aliquoted and maintained at −70 °C.

### 2.2 ELISA assays to measure total, oligomeric alpha-synuclein and hemoglobin levels

Total alpha-synuclein, oligomeric alpha-synuclein and hemoglobin levels were estimated by means of enzyme-linked immunosorbent assay (ELISA) (Human alpha-synuclein ELISA kit (Invitrogen, USA), Human Synuclein OLIGO kit (ajRoboscreen, Germany) and Hemoglobin (Human) ELISA kit (Abnova, USA), accordingly), as described previously [2], [3], [4]. Levels of oligomeric alpha-synuclein were estimated by means of ELISA with 5G4 antibodies designated for detection of alpha-synuclein oligomers in human fluid samples [5]. Undiluted plasma samples were examined in case of alpha-synuclein measurements. For hemoglobin level estimation plasma samples were diluted according the manufacturer׳s recommendations. In all ELISA assays each sample was measured in triplicate.

### 2.3 Statistical analysis

To assess differences between groups, the Mann–Whitney test was used, and the correlations were evaluated by exponential regression analysis. The correlations were evaluated using the Spearman correlation coefficient. Sex variables were analyzed by Chi-square and Fisher׳s exact tests. Experimental data are given as median (min–max). The level of significance was set at *p*<0.05. Statistical analysis was carried out using SPSS 12.0.

## Figures and Tables

**Table 1:** Alpha-synuclein (total, oligomeric) levels in iPD patients and controls.

	iPD patients	Controls	*p*
Total alpha-synuclein, (median (min–max)), ng/ml	3.89 (0.15–51.11)	3.88 (1.26–23.24)	0.478
Oligomeric alpha-synuclein, (median (min–max)), pg/ml	4.14 (0.73–40.9)	5.42 (1.05–129.19)	0.766
Oligomeric/total alpha-synuclein (median (min–max)), %	0.15 (0.006–2.033)	0.13 (0.01–6.63)	0.692
